# The effects of endogenous and exogenous androgens on cardiovascular disease risk factors and progression

**DOI:** 10.1186/1477-7827-7-44

**Published:** 2009-05-12

**Authors:** Panagiota Manolakou, Roxani Angelopoulou, Chris Bakoyiannis, Elias Bastounis

**Affiliations:** 1 Department of Histology and Embryology, Medical School, National and Kapodistrian University of Athens, Greece; 2 First Department of Surgery, National and Kapodistrian University of Athens Medical School, Laiko Hospital, Athens, Greece

## Abstract

Cardiovascular disease incidence rates have long been known to significantly differ between the two sexes. Estrogens alone fail to explain this phenomenon, bringing an increasing amount of attention to the role of androgens. Contrary to what was initially hypothesized, androgens seem to have an overall cardioprotective effect, especially in men. Recent studies and published data continue to support this notion displaying a consistent inverse correlation with atherosclerosis progression and cardiovascular disease both in regressive and prospective study models. Clinical studies have also revealed what seems to be a differential androgenic effect on various cardiovascular risk factors between men and women. Further insight indicates that in order to avoid confusion it may be also preferable to separately examine the effects of endogenous androgen levels from exogenous testosterone administration, as well as discern the differential results of low to normal and supraphysiological administration doses. This review summarizes old and recent data according to the above distinctions, in an attempt to further our understanding of the role of androgens in cardiovascular disease.

## Background

Cardiovascular disease is characterized for displaying a significant difference in incidence rates between men and women. Even after adjusting for all major risk factors, men have a two-fold risk to die from coronary heart disease compared to women, a fact that remains consistent across various ethnic and social groups [1]. It was initially attempted to attribute this phenomenon to a possibly protective effect of estrogens in the female vasculature. However, there are several factors arguing against this particular theory. For example, a more careful study of the epidemiology of cardiovascular disease reveals that incidence rates in women do not follow the trend of other estrogen-related diseases, which is to say that they do not seem to display a distinct break-point after menopause [2]. In addition, the ongoing debate over the effects of hormone replacement therapy (HRT) on cardiovascular events in women [3-5] has further weakened the belief that estrogens act as the major determinants of gender-specific differences in cardiovascular event rates. As a result, researchers in recent years have turned with increasing interest on androgens and the roles that they can play.

Androgens, like most hormones, are partly difficult to study due to their physiological concentration variability. Their serum levels display a circadian and circannual variation [6]. Furthermore, their levels display a steady reduction with age in both men and women [7-14] which can act as a confounding factor and has prompted most studies to include age-adjusted multiple linear regression analysis for their data. Despite these details however, it is becoming increasingly apparent that androgens are inherently influential in many aspects of cardiovascular disease, including both primary pathophysiological mechanisms and contributing major risk factors.

The aim of this review is to summarize recent findings on the effects of androgens on most of the aforementioned components of cardiovascular disease and highlight points of interest that can help shed some light on the controversial findings regarding sex steroids and their effects on the human heart and vasculature.

### Androgen levels in men with a history of heart disease

The most obvious starting point in the quest for examining the role of androgens on cardiovascular disease is looking for differences in androgen levels between healthy men and men that already have a history of heart disease. Based on the assumption that androgens have a deleterious effect on the male cardiovascular system, researchers from as early back as 1979 had tried to detect differences in testosterone levels in men that had recently presented with acute myocardial infarction. Surprisingly enough, it was soon noted that testosterone levels tended to be normal prior to infarction, decreased shortly afterwards and normalized approximately a year later [15].

Later reviews of published data continue to mention that in almost half of the studies examined, men with a history of a coronary artery disease had a higher prevalence of hypoandrogenemia [16-18]. It was thus assumed that low testosterone levels can be considered either a protective factor against myocardial infarction, or a resulting effect from the stress an acute myocardial infarction submits the male organism to.

The possibility of a hitherto unknown protective androgen effect against acute cardiovascular events was initially met with enthusiasm, but a number of prospective cohort studies quickly dashed that hope since none of them could establish the predictive value of testosterone levels for myocardial infarction or stroke after adjusting for other relevant confounding factors [16,17].

The alternative option that low testosterone levels might be a consequence of stress was thus examined more closely. However, this theory could not explain the fact that testosterone levels remained low several months after the occurrence of a myocardial infarction. Furthermore, while one older study among 55 men claimed that testosterone and free testosterone inversely correlated with the degree of coronary disease after adjusting for age and BMI (body mass index) [18], a more recent one among 90 patients [8] and another among 403 men [12] found no such association. What is even more impressive is that, according to English *et al*, none of their 17 control patients that suffered from severe valvular disease, and were thus under heavy stress from a non-atherosclerotic heart condition, displayed similarly low levels of testosterone. If hypotestosteronemia could be attributed to stress, then one would at least expect testosterone levels to further decrease in more severe cases of coronary disease, which apparently is not the case. Hopefully future research will provide more insight into this small conundrum.

### Endogenous androgen levels and the progression of atherosclerosis

Since looking into the level differences in men with an already existing positive history of heart disease could yield no conclusive conjecture, researchers shifted their focus to a more progressive approach of the effects of androgens in the cardiovascular system. Thus, a large number of clinical studies have attempted to investigate the possible effects of endogenous androgen levels on the progression of various atherosclerotic indices, aspiring to reveal their involvement in a more ongoing setting. Unlike in other aspects of androgen action in the vasculature, the resulting data here seem to be more consistent, attributing androgens with a clearly positive influence on atherosclerotic outcomes.

Carotid artery intima-media thickness (IMT) estimated via carotid ultrasonography is one of the most popular markers used to investigate the progression of atherosclerosis. A number of studies focused on correlating carotid ultrasonography results with androgenic hormone measurements seem to have reached similar conclusions. In all cases, subjects located in the higher tertiles for testosterone levels corresponded with statistically significant lower IMT values compared to subjects in the lower testosterone quartiles. Interestingly enough, this finding persisted in both genders and was consistent in all patient groups, including both premenopausal and postmenopausal women, as well as young and old male adults (see Table 1).

**Table 1:** Various studies continue to support that endogenous androgen levels are inversely correlated with the progression of atherosclerosis.

	Androgen effects in men	Androgen effects in women
Coronary artery atherosclerosis	Negative correlation [8,27]	
Carotid artery atherosclerosis	Negative correlation [12,14,22-25]	Negative correlation [7,10,19-21]
Aorta atherosclerosis	Negative correlation [13,26]	Positive correlation (*largely diluted when adjusted for other cardiovascular risk factors*) [13]
Peripheral vasculature atherosclerosis	Negative correlation [24]	

In spite of the fact that androgens are not specific for the female gender, or perhaps because of it, their influence in this has been studied in women as well as men. However, there are few studies examining the effects of endogenous androgen levels in premenopausal women. Bernini *et al *observed that, while androstenedione and free testosterone seem to be significantly correlated with carotid IMT measurements even after adjusting for age and other cardiovascular factors in postmenopausal women [7,10], this correlation fails to shine through in the group of premenopausal women [7]. Other studies focused on postmenopausal subjects either agreed with these findings [19] or stressed the importance of total testosterone instead of androstenedione and free testosterone [20,21], (see Table 2). None of them however disputed that androgenic hormones inversely correlate with carotid atherosclerosis progression.

**Table 2:** An overview of the statistically significant negative correlations from various recent study results regarding the effects of endogenous androgen levels on carotid IMT measurements in women.

Study	Subjects	DHEA-S	A	FT	TT
Bernini *et al*,1999 [7]	101 women	SS	SS	SS	-
	- 48 postmenopausal	-	SS	SS	-
	- 53 premenopausal	-	-	-	-
Kiechl *et al*,2000 [9]	379 women	-			
Bernini *et al*, 2001 [10]	44 postmenopausal women	-	SS	SS	-
Debing *et al***, **2007 [19]	56 postmenopausal cases and 56 postmenopausal controls	-	SS	SS	SS
Golden *et al*, 2002 [20]	182 postmenopausal cases and 182 postmenopausal controls	-	-		SS
Montalcini *et al***, **2007 [21]	101 non-obese postmenopausal women				SS

DHEA-S = dehydroepiandrosterone sulfate, A = androstenedione, FT = free testosterone, TT = total testosterone, SS = statistically significant, - = no correlation or not-statistically significant correlation

Similarly in men, there are several studies that concur in the protective effect of androgens in the progression of atherosclerosis and almost all of them indicate either free serum testosterone or total serum testosterone levels as the major determining factor, even after adjusting for age and other risk factors (see Table 3). These include both population-based cross-sectional [12,22-25] and progressive studies [14], further strengthening their conclusions.

**Table 3:** An overview of the statistically significant negative correlations from various recent study results regarding the effects of endogenous androgen levels on carotid IMT measurements in men.

Study	Subjects	DHEA	DHEA**-**S	FT	TT
Kiechl *et al*, 2000 [9]	371 men		-		
Van de Beld *et al*, 2003 [12]	403 elderly men	-	-		SS
	- 139 with CVD	-	-		SS
	- 263 without CVD	-	-		SS
Muller *et al*, 2004 [14]	195 elderly men			SS	-
De Pergola *et al*, 2003 [22]	127 overweight and obese young men			SS	
Makinen *et al*, 2005 [23]	96 andropausal and 140 normal elderly men				SS
Yang *et al*, 2005 [24]	70 elderly men (38 cases and 32 controls)			SS	-
Svartberg *et al*, 2006 [25]	1482 men with or without CVD		-	-	SS

DHEA = dehydroepiandrosterone, DHEA-S = dehydroepiandrosterone sulfate, FT = free testosterone, TT = total testosterone, SS = statistically significant, - = no correlation or not-statistically significant correlation

While carotid IMT measurements are indeed very popular, a few studies have focused on atherosclerotic manifestations of the aorta instead. Demirbag *et al *measured the thoracic aorta intima thickness (TAIT) via transesophageal echocardiography (TEE) and validated its negative correlation with total serum testosterone [26]. Hak *et al *investigated mean hormonal levels in relation to aortic atherosclerosis using radiographic detection of calcified deposits in the abdominal aorta as an indicator of the presence of atherosclerotic lesions [13]. Among the 1032 subjects involved (504 men and 528 women), no association was made to dehydroepiandrosterone sulfate (DHEA-S) levels. There was however a clearly negative correlation between severe atherosclerosis and both bioavailable and total testosterone levels in men, which remained even after adjusting for other cardiovascular risk factors. Among women on the other hand, both bioavailable and total testosterone levels seemed to positively correlate with aortic atherosclerosis, but this correlation was diluted after adjusting for other cardiovascular risk factors. This could perhaps be linked to the adverse effect of androgens on the lipid profile in women.

Likewise, English *et al *focused on coronary angiogram results [8], making comparisons between men with ischaemic heart disease (> 75% occlusion in at least one of the major coronary arteries) and control subjects that were either healthy or presented with non-atherosclerotic forms of heart conditions in total a sample of 90 men. This study also indicated a negative correlation between free and bioavailable testosterone, as well as the free androgen index (FAI). After adjusting for age and other risk factors, only the bioavailable testosterone and FAI correlations persevered. These findings were somewhat confirmed by Philips *et al*, who investigated coronary artery disease in men that had not previously exhibited acute ischaemic manifestations and in whom testosterone and free testosterone levels were found to negatively correlate with the angiography findings after adjusting for age and body mass index (BMI) [27].

The fact remains however that we have yet to determine which of the aforementioned hormonal measurements presents with the strongest or more consistent correlation to any of the above atherosclerotic markers. While all remained in agreement of the protective effect of androgens in the progression of atherosclerosis, each of the aforementioned studies indicated a different androgenic hormone measurement as significantly associated with its results. This may be attributed to the variability of each of the patient samples or the array of risk factors used to adjust these findings and the large divergence in study designs. It still remains though something that we should further clarify if we ever plan to use one of these measurements both for research and practical purposes.

### Androgens and the vascular tone

Although different in its nature from atherosclerotic progression per se, the regulation of endothelial function and vascular tone in general remain an important component of vascular physiology and predictor of cardiovascular disease. The mechanisms underlying the pathophysiology of impaired vascular tone regulation and thus the emergence of arterial hypertension are very complex and beyond the scope of this study. It is nevertheless important to attempt to summarize the more important androgen effects that have been recently reported.

According to a longitudinal study among 206 men, testosterone levels measured 5–10 years prior proved to be an independent negative predictor factor for arterial stiffness after adjusting for other risk factors [28], an early predictor for systolic hypertension. Testosterone has similarly been reported to bear both acute and chronic vasodilatory effects upon various vascular beds. Studies from as early back as the 1940s report the use of testosterone in order to improve angina pectoris in men with coronary artery disease [29]. Indeed, when infused directly into the left coronary artery, testosterone induces vasodilation that improves the existent coronary flow [6] even if it requires high supra-physiological doses [30]. Other studies report that acute and chronic administration of testosterone in either physiological or supraphysiological doses can improve exercise-induced ST-segment depression on the ECG of men with established coronary disease [31]. Animal model studies also exhibited the same dose-dependent vasodilatory effect on not only isolated coronary, but also femoral and pulmonary arteries [6].

The rapid onset of testosterone's acute vasodilatory action and the fact that it was not abolished by flutamide, an AR (androgen receptor) blocker [6], led to the theory that this particular effect was not mediated through the AR and its genomic signaling pathway. This was enhanced by later data that supported that testosterone's vasodilatory action was not significantly altered in Tfm (testicular feminized) mice that express a non-functional AR [32].

The same study however also reported that the added effect of AR-deficiency and low testosterone levels in Tfm mice led to impaired endothelial function and voltage-operated calcium channel (VOCC) activity. While this argues against the release of endothelium-derived products or the inhibition of VOCCs as the underlying mechanism for testosterone's acute vasodilatory action, it does have implications for the development of systemic arterial hypertension.

In concordance with this, another study discovered that congenital hypogonadal men displayed distinctly impaired vascular reactivity in resistance arteries, as opposed to conduit arteries, due to reduced endothelium-dependent (via reduced NO availability) and endothelium-independent vasodilation [33]. What is more impressive is that prolonged testosterone replacement therapy in those men further impaired endothelium-dependent vasodilation by completely removing all available NO. This is in full disagreement with its effects in eugonadal men, which means that more research is needed in order to better understand the way that chronic testosterone deprivation impairs vascular tone regulation.

### Associating androgens with various risk factors

In addition to their effects on the progression of atherosclerosis in general, clinical studies have also attempted to separately investigate the relationship between androgens and some of the major risk factors that are often being adjusted for. These mostly include cross-sectional studies that attempt to correlate various hormone levels with a wide set of well established cardiovascular risk factors. To avoid any confusion, these should be differentiated from studies that investigate exogenous testosterone administration or special conditions which result in supraphysiological endogenous androgen levels, since it has been argued that the effects of androgens may vary in these cases.

It is mostly agreed upon that androgens have a favorable effect on cardiovascular risk factors in men [1,16]. Newer studies continue to reaffirm this observation. One obvious example is the make-up of a patient's lipid profile. Although not always reaching the threshold of statistic significance, a variety of recent studies among men with or without a history of cardiovascular disease continue to present data on the positive effect of androgens on the male lipid profile, mainly associating total testosterone measurements and the free androgen index with HDL (high-density lipoprotein) and triglyceride levels [11,25] (see Table 4). Similar data also exist for other risk factors, such as high arterial blood pressure and diabetes. These, however, apparently also display significant correlation with dehydroepiandrosterone (DHEA) and dehydroepiandrosterone sulfate (DHEA-S) in a similarly protective fashion [9,13,25,34] (see Table 4).

**Table 4:** The effect of androgens on the lipid profile and other risk factors in men.

	Tchole	HDL	LDL	TG	Blood pressure	Postload insulin	Diabetes
DHEA	- [34]	- [34]			↓ [34]		↓ [34]
DHEA**-**S	- [13,34]	- [13,34]			↓ [34] - [13]	- [13]	↓ [9,34] - [13]
T	- [11,13]	- [11,13] ↑ [25]	- [11]	↓ [11,25]	↓ [25] - [11–13]	↓ [13]	- [12,13]
FAI	- [11]	↓ [11]	- [11]	↓ [11]	- [11]		

Tchole = total cholesterol, TG = triglycerides, DHEA = dehydroepiandrosterone, DHEA-S = dehydroepiandrosterone sulfate, T = testosterone, FAI = free androgen index, ↑ = positive correlation, ↓ = negative correlation, - = no correlation or not statistically significant correlation

Unfortunately, the same protective effect does not extend to the female population. Contrary to men, higher endogenous testosterone levels have been claimed to have an adverse effect on women's lipid profile [16]. Indeed, newer studies seem to be in agreement, since total serum testosterone levels and FAI in particular have been associated with higher total cholesterol, LDL cholesterol and triglycerides on one hand, and lower HDL cholesterol levels on the other [19,35,36] (see Table 5). Furthermore, although lacking an adverse correlation, the androgen protective effect regarding arterial blood pressure and diabetes fails to shine through among female subjects [9,10,13,19,37] (see Table 5).

**Table 5:** The effect of androgens on the lipid profile and other risk factors in women.

	Tchole	HDL	LDL	TG	Blood pressure	Postload insulin	G/I	Diabetes
DHEA**-**S	- [10,13,35,36]	- [10,13,35,36]	- [10,35,36]	- [35,36]	- [10,13,36]	- [13]	- [10]	- [9,13]
FT	- [10]	- [10]	- [10]	- [19]	- [10,19]		- [10]	- [19]
TT	- [10,13,35] ↑ [36]	- [10,13,35] ↓ [36]	- [10,35] ↑ [36]	- [35] ↑ [19,36]	- [10,13,19,36]	- [13]	- [10]	- [13,19]
FAI	- [35] ↑ [35*,36] (*patients only)	↓ [35,36]	↑ [35,36]	- [35] ↑ [35*,36] (*patients only)	- [36]			

Tchole = total cholesterol, TG = triglycerides, G/I = fasting glucose/insulin ratio, DHEA-S = dehydroepiandrosterone sulfate, A = androstenedione, FT = free testosterone, TT = total testosterone, FAI = free androgen index, ↑ = positive correlation, ↓ = negative correlation, - = no correlation or not statistically significant correlation

An interesting observation at this point is the apparent effect of androgens on Lp(a) (lipoprotein a). Lp(a) is considered an independent risk factor for cardiovascular disease and is distinctly known for its strong genetic component. Admittedly, at least one study among women has failed to establish a relationship between endogenous testosterone concentrations and Lp(a) levels [36]. We should note however that GnRH (gonadotrophin-releasing hormone) antagonists or GnRH agonist administration in doses that artificially suppress androgen production in men can apparently lead to a severe Lp(a) increase [16,17]. A similarly inverse relationship has also been shown between androgens and plasminogen activator inhibitor-1 (PAI-1), fibrinogen and other prothrombotic factors [16,17,38], rounding up the cardioprotective effects of endogenous androgens on the major components of the basic risk profile for heart disease.

Androgens are further related to another very significant cardiovascular risk factor: obesity. Morbid obesity in men has been associated with decreased androgen levels, while serum testosterone levels in general inversely correlate with visceral fat mass [16,17,29,38]. These observations went hand in hand with the implication of low androgen levels in insulin resistance, setting the prerequisites for associating androgens with the metabolic syndrome. It has yet to be determined however whether the relationship described is one of cause or effect. Some have argued that many free testosterone concentration measurements use a tracer analogue method that is affected by SHBG (sex hormone-binding globulin) levels, which in turn are decreased by obesity and high insulin concentrations [17,29,38]. Others claim that low androgen levels in obese men can be attributed to increased peripheral aromatization of androstenedione to estrone by the aromatase enzyme [16]. According to at least one study though, decreased testosterone levels in men with high body mass index coexisted with significantly decreased estradiol levels, contradicting the latter theory [8]. And most importantly, induction of hypotestosteronemia through the administration of GnRH (gonadotropin-releasing hormone) agonists has been shown to cause an increase in body fat mass, establishing the influential role of androgens in the settings of male obesity.

Indeed, most if not all recent studies confirm that DHEA-S, free serum testosterone, total serum testosterone and FAI inversely correlate with not only BMI, but also waist-to hip ratio (WHR) and total body fat mass measurements in men [8,10,11,13,22,34] (see Table 6). Similar correlations have also been noted in women, although their statistic significance is less evident in the female sex [10,13,37]. The matter of female obesity and androgens however may still need in-depth research, since observations made in women with polycystic ovaries syndrome (PCOS) contradict the previous statement [16,17]. The coexistence of hyperandrogenemia with obesity and impaired insulin sensitivity seems telling, but we have yet to determine whether high androgen levels in themselves can be considered responsible for obesity and insulin resistance, or if insulin resistance and the resulting high insulin concentrations reinforce hyperandrogenemia by stimulating androgen production in the ovaries. In any case, one should keep in mind that testosterone concentrations in women with PCOS far surpass the physiological range and should probably be examined separately from the effects of low to normal endogenous testosterone levels in the general population.

**Table 6:** Linking androgen levels with obesity and body fat distribution

	**BMI**		**WHR**		**BF**	
	Men	Women	Men	Women	Men	Women
DHEA	↓ [13,34]		↓ [34]			
DHEA**-**S	↓ [34]	- [13] ↓ [10]	- [13] ↓ [34]	- [10] ↓ [13]		
A		↓ [10]		↓ [10]		
FT	↓ [22]	- [10]		- [10]	↓ [22]	
TT	↓ [8,11–13,25]	- [10] ↓ [13]	- [13]	- [10,13]	↓ [11]	
FAI	↓ [11]			↑ [37]	↓ [11]	

BMI = Body Mass Index, WHR = Waist-to-Hip Ratio, BF = Body Fat mass, DHEA = dehydroepiandrosterone, DHEA-S = dehydroepiandrosterone sulfate, A = androstenedione, FT = free testosterone, TT = total testosterone, FAI = free androgen index, ↑ = positive correlation, ↓ = negative correlation, - = no correlation or not statistically significant correlation

Such an obvious link between androgens and obesity however raises another interesting question. Could we perhaps attribute the relationship between androgens and other risk factors, such as serum lipid concentrations and insulin sensitivity, to their effects on body fat mass? It seems entirely logical that lower BMI and WHR measurements might conclude in a more favourable lipid profile, acting as a confounding factor [39]. However, one might claim that if that were the main underlying mechanism, then for all intents and purposes a similar effect should also appear on the female lipid profile, since androgens within a normal range inversely correlate with BMI in women as well. Furthermore, there is evidence that endogenous androgen levels continue to display the same correlations with serum lipid levels even after adjusting for BMI [11]. In other words, the evidence suggest that, despite their link to total body fat mass, the main effect of androgens is achieved through an independently influential role.

Finally, an interesting novel notion has recently turned scientific interest towards the investigation of the possible immunomodulating effects of testosterone [1]. Yang *et al *attempted to study the link between androgens and various cytokines among other things in elderly men, and managed to confirm a statistically significant inverse correlation between free serum testosterone levels and CRP (C-reactive protein), IL-6 (interleukin-6) and sICAM-1 (soluble intercellular adhesion molecule 1) [24]. This, confirmed by studies on the implication of androgens in inflammatory response on a molecular level, indicates a new field of research concerning their actions in atherosclerosis.

### Implications for androgen treatment

When looking into the effects of androgens on cardiovascular disease, exogenous testosterone treatment should be examined separately from the effects of its endogenous counterpart in order to avoid confusion. The reason for this is that exogenous intervention seems to wield different results from the spontaneous fluctuation of endogenous androgen levels. As a result, despite the clearly cardioprotective actions of androgens in men, if not in women as well, the matter of testosterone supplementation is one that requires further research on its own.

Another important point we need to stress is the necessity to differentiate between testosterone administration in low-normal and supraphysiological doses, as well as between hypogonadal and eugonadal men. For example, when examining the effects of testosterone administration on plasma lipids, it becomes apparent that supraphysiological doses of androgens lower HDL concentrations and mildly increase LDL and total serum cholesterol [16,29,38]. The same applies for anabolic steroid abuse [16,40]. Most studies however report that physiological testosterone replacement in hypogonadal men does not change or only mildly lowers HDL concentration and usually has no effect or mildly decreases LDL or total serum cholesterol [29,30,38,41]. In addition, despite its strongly genetic component, testosterone supplementation has also been shown to decrease serum levels of Lp(a) in men [16,29].

Both testosterone administration and anabolic steroid use seem to decrease PAI-1 and fibrinogen in men and women, attesting to androgens' anticoagulatory and fibrinolytic effects [16,17,29,40]. Things are not as clear however with serum inflammation markers. A study of testosterone administration in 65 eugonadal men reports no correlation with circulating CRP levels [41]. Another study of DHT (dihydrotestosterone) treatment in 33 men with partial androgen deficiency also reported no changes in either CRP, sICAM-1 or sVCAM-1 serum levels [42]. On the other hand, in a study of gonadotropin treatment among 29 men with hypogonadotropic hypogonadism, all inflammation markers measured showed a distinct decrease [43]. These results should be interpreted with care though, since gonadotropin administration in these men is not the same as exogenous testosterone administration and its effects may fall within the category of the effects of endogenous testosterone levels.

It has already been mentioned that obesity has been associated with decreased testosterone levels, a relation that was initially attempted to be attributed to either increased peripheral aromatization to estrogens or decreased SHBG concentrations. Further attesting against these two theories is the observation that testosterone replacement in hypogonadal men can effectively reduce whole body fat mass in a dose-dependent manner [1,16,29,38]. Even in eugonadal men testosterone treatment can have a positive effect on visceral fat mass. It can also improve insulin sensitivity, but care should be taken to distinguish between the effects of low and high testosterone doses on insulin resistance. Testosterone administration can apparently improve insulin sensitivity and reduce insulin levels in the case of physiological supplementation, but it can also lead to increased insulin resistance when attempted in a higher dosage [29,38]. The implication of anabolic steroids in increased insulin resistance and altered glucose tolerance seems to fall within the latter category [40]. As a side observation, it is interesting to note that a feedback relationship between insulin and testosterone levels may exist as well. Insulin infusion in obese men can increase testosterone levels, while increased insulin levels have been shown to contribute to increased androgen production in women with PCOS [16,17].

The matter of exogenous androgen administration in women can also prove fairly interesting. A 20-year observation study of testosterone administration among 293 female-to-male transsexuals reported no increase in cardiovascular deaths [44] even though they received considerably high testosterone doses. On the other hand, a recent study among 513 postmenopausal women, 25 of which reported intramuscular administration of testosterone along the lines of hormone replacement treatment, found a marked increase in severe aorta atherosclerosis among those that had received testosterone treatment [45]. Although there is the distinct difference that testosterone treatment in the latter study coincided with estradiol administration which may act as a confounding factor, the fact remains that, despite its beneficiary effects in men, testosterone supplementation should be examined separately in women.

Finally, it should also be mentioned that Dougherty *et al *attempted to investigate an alternative option to outright testosterone treatment: the administration of anastrozole, an oral aromatase inhibitor. Anastrozole was able to normalize testosterone levels in hypogonadal men and reduce estradiol levels. Contrary to testosterone treatment however, no significant changes in either lipid profile or inflammation markers were detected, which was partly attributed to the mixed effects of increasing testosterone and reducing estradiol levels [46].

## Conclusion

After the initial disregard concerning their role in cardiovascular disease, androgens are now increasingly attracting scientific interest. A large array of data testifies in favor of a variety of cardioprotective androgen effects in men mostly, but in many cases in women as well. Different endogenous androgenic hormone measurements have been associated with the progression of atherosclerosis and an inverse correlation was almost always displayed in both men and women. On the other hand, total serum testosterone levels and the free androgen index seem to differentially associate with various risk factors in men and women, which mainly consist of various parameters of the lipid profile, arterial blood pressure, diabetes and obesity. Following the verified existence of interacting mechanisms at the molecular level, certain associations between androgens and circulating inflammatory markers have been established as well.

In order to avoid confusion, exogenous testosterone treatment should be examined separately from the effects of endogenous androgen levels. The same applies for different levels of administration doses. Testosterone supplementation in low to normal levels in hypogonadal men has mostly been shown to benefit the subjects receiving it, although not to the degree perhaps one would expect from the many positive effects of its endogenous counterpart. Testosterone administration in supraphysiological doses however, along with anabolic steroid abuse, seems to adversely affect both the lipid profile and insulin sensitivity in men. Its effects in women have yet to be researched in depth, while an attempt to replace testosterone treatment with oral aromatase inhibitors failed to produce any significant results.

In conclusion, it can be claimed beyond doubt that androgens play an active role in the appearance and progress of cardiovascular disease. As the debate on the role of estrogens continues, understanding the minutiae of the implication of androgens becomes increasingly important, while by attempting to further clarify the details of that implication we can also understand the mechanisms underlying cardiovascular manifestations in general.

## Abbreviations

HRT: hormone replacement treatment; BMI: body mass index; IMT: intima-media thickness; TAIT: thoracic aorta intima thickness; TEE: transesophageal echocardiography; DHEA-S: dehydroepiandrosterone sulfate; FAI: free androgen index; HDL: high-density lipoprotein; DHEA: dehydroepiandrosterone; Lp(a): lipoprotein a; GnRH: gonadotrophin-releasing hormone; PAI-1: plasminogen activator inhibitor-1; SHBG: sex hormone-binding globulin; WHR: waist-to hip ratio; PCOS: polycystic ovaries syndrome; CRP: C-reactive protein; IL-6: interleukin-6; sICAM-1: soluble intercellular adhesion molecule 1; AR: androgen receptor; Tfm: testicular feminized; DHT: dihydrotestosterone.

## Competing interests

The authors declare that they have no competing interests.

## Authors' contributions

PM selected all reference material and drafted the initial manuscript. Consequently all authors contributed equally in the development of the article's viewpoints and its critical revisions. All authors read and approved the final manuscript.
